# Effect of Maternal Age on Hippo Pathway Related Gene Expressions and Protein Localization Pattern in Human Embryos

**DOI:** 10.22074/cellj.2020.6860

**Published:** 2020-09-08

**Authors:** Sahar Gharanfoli, Abdolhossein Shahverdi, Azam Dalman, Pooneh Ghaznavi, Hiva Alipour, Poopak Eftekhari-Yazdi

**Affiliations:** 1. Department of Developmental Biology, University of Science and Culture, Tehran, Iran; 2. Department of Embryology, Reproductive Biomedicine Research Center, Royan Institute for Reproductive Biomedicine, ACECR, Tehran, Iran; 3. Biomedicine Group, Department of Health Science and Technology, Faculty of Medicine, Aalborg University, Aalborg, Denmark

**Keywords:** Embryonic Development, Hippo Signalling, Maternal Age

## Abstract

**Objective:**

The Hippo pathway plays an important role in embryo development, and separation of trophectoderm (TE) and inner cell mass (ICM) cell lines. Therefore, this study investigated effect of maternal age on activity of Hippo pathway in human embryos.

**Materials and Methods:**

In this experimental study, the developed up embryos to the blastocyst stage and the embryos whose growth stopped at the morula stage were collected from women aged 20-30 years old (young group, 94 embryos) and >37 years (old group, 89 embryos). Expression of OCT4, SOX2, CDX2, GATA3, YAP genes and the relevant proteins, in the both groups were evaluated using respectively quantitative reverse transcription-polymerase chain reaction (qRT-PCR) and immunofluorescence methods.

**Results:**

There was no significant difference in the expression level of OCT4, SOX2, CDX2, GATA3 and YAP genes in blastocyst and morula stages, between the two groups. However, SOX2 and CDX2 gene expressions in morula stage embryos of the old group was statistically lower than that of the young group (P=0.007 and P=0.008, respectively). Additionally, in the embryos collected from women with >37 years of age, at the blastocyst stage, phospho-YAP (p-YAP) protein was found to be accumulated in the TE, but it was almost disappeared from the ICM. Additionally, in the old group, contrary to the expectation, YAP protein was expressed in the ICM, rather than TE.

**Conclusion:**

The results of this study showed that YAP and P-YAP among the Hippo signalling pathway may be altered by increasing age.

## Introduction

Pre-implantation development starts from fertilization and continues with repeated divisions. It leads to the formation of a complex structure called blastocyst (1, 2). The major developmental events are embryo cleavage, compaction and polarization, trophectoderm/inner cell mass (ICM) specification and blastocyst formation with two different cell lines (3, 4). Trophectoderm (TE) plays an important role in implantation as it interacts with the mother’s uterus and participates in the formation of placenta (5). After implantation, ICM forms three germ layers, ultimately generating all tissues of the body (6, 7). Several mechanisms including FGF, Erk/Mapk and Hippo signalling pathways are involved in this differentiation process (8, 9). Hippo signalling pathway, along with the inside-outside axis of embryo, regulates cell-fate switch by modulating transcription factor Tead4 activity (10, 11). While Tead4 is active in outside-cells and promotes TE development, it is suppressed in inside-cells by cell contact- and phosphorylation-mediated inhibition of nuclear localization of the Tead4 coactivator Yes-associated protein (YAP) (12-14).

In the outer cells of embryo, Hippo signalling is likely suppressed by another signal which is produced by the cell polarity. YAP is translocated to the nucleus, where it increases *CDX2* and *GATA3* gene expressions together with TEAD4, which in turn induces trophoblast formation and *OCT4* downregulation (12, 13, 15). In the ICM, where Hippo signalling is active, YAP is phosphorylated by LATS. Therefore, YAP remains in the cytoplasm. In the absence of a nuclear YAP, since TEAD4 remains inactive, *CDX2* and *GATA3* genes are not expressed and the initial expression of *OCT4* is maintained (16, 17). Many factors -such as culture medium, oxygen concentration, developmental stage and age- can affect developmental gene expression and likely Hippo signalling pathway. Mantikou et al. (18) in 2015 investigated the effect of women’s age on gene expression in three groups of ≤ 35, 36-38, and ≥39 years old. The results showed significant differences between the three groups. Since the expression of developmental genes are under the control of various signalling pathways, in this study, effect of women’s age on activity of the Hippo signalling pathway was investigated. According to a previous study performed by Bellieni et al. (19) in 2016, the woman’s optimal age for reproduction is 20- 30 years, regarding that the number of oocytes is decreased in women after ≥35 years of age. In the present study, due to the importance of Hippo signaling pathway in the embryo development, and separating TE and ICM cell lines, effect of women’s age on Hippo signalling pathway activity of human embryos was investigated.

## Materials and Methods

This experimental study was in accordance with the Declaration of Helsinki, following the approval of Ethical Committee of Royan Institute (Tehran, Iran; approval number: IR.ACECR.ROYAN.REC.1395.8). The patients were selected based on age, body mass index (BMI), cause of infertility, type of control ovarian stimulation (agonist and antagonist), Number of *in vitro* fertilization/intracytoplasmic sperm injection (IVF/ICSI) procedure as well as the oocyte and embryo quality, ovarian reserve and number of treatment cycles. Inclusion criteria for the study encompass the quality of embryo, women’s age and lack of genetic diseases.

Exclusion criteria include embryos of the patients which were not developed to morula and blastocyst stages. Fresh and frozen embryos from consenting couples attending Royan Institute for infertility treatment (after signing written consent) were cultured in G_1_V_5_^TM^ medium (Vitrolife, Sweden) for three days, before transferring to the G_2_V_5_^TM^ medium (Vitrolife). The embryos (either those stopped at the morula stage or those reaching the blastocyst stage) were collected on day 5 and divided into two groups: the embryos collected from women aged 20-30 (young group) and the embryos belonging to women >37 years of age (old group).

### Gene expression analysis

Total RNA from embryos that either reached to the blastocyst stage or stopped at the morula stage, were extracted using the RNeasy micro Kit (Qiagen, Germany), before, synthesizing complementary DNA (cDNA) by Fermentase kit (Germany) (20). PCR reaction was run as follow in a total volume of 25 μl: master mix (10 μl, Thermo, USA), forward and reverse specific primers (each 1 μl), cDNA (1 μl) and nuclease free water (12 μl). quantitative reverse transcription polymerase chain reaction (qRT-PCR) protocol was carried out using SYBER Green (Takara, Japan) according to the following program: initiation step at 94˚C for 300 seconds; 35 cycles at 94˚C for 40 seconds followed by 60˚C for 40 seconds and 72˚C for 40 seconds, terminated by incubating at 72˚C for 10 seconds. For presentation of data, products specificity was confirmed by melt curve analysis. Then, the qRT-PCR results were estimated using 2^-ΔΔCt^ formula. Gene expression analysis was performed for pluripotency markers (*OCT4*, *CDX2*, *GATA3* and *SOX2*) and Hippo signalling marker (*YAP*). *GAPDH* was considered as housekeeping gene. The oligonucleotide primers were designed using NCBI site, Perl Primer and Gene Runner software. The utilized primers in the present work are listed in Table 1.

### Immunocytochemical analysis

The embryos were fixed in 4% paraformaldehyde solution (Merck, Germany) for 15 minutes at room temperature and placed in 0.25% Triton X-100 (Sigma, USA) soluble in phosphate-buffered saline (PBS, Gibco, USA) for 30 minutes at room temperature. Then, the embryos were incubated with 5% BSA in donkey serum for 60 minutes at room temperature to block unspecific binding of the antibodies, before being incubated with the following primary antibodies: OCT4 (mouse monoclonal; Santa Cruz, USA, 1:100 dilution), CDX2 (Goat polyclonal IgG, Santa Cruz, USA, 1:100 dilution), YAP (Rabbit polyclonal; Proteintech, USA, 1:100 dilution) or P-YAP (Rabbit polyclonal; Abcam, UK, 1:100 dilution) overnight at 4˚C, as previously described (6). Incubation was continued with conjugated donkey antirabbit IgG (Invitrogen, USA, 1:600 dilution) for YAP and P-YAP, donkey anti-mouse IgG (Invitrogen, USA, 1:600 dilution) for OCT4 and donkey anti-goat IgG (Invitrogen; 1:600 dilution) for CDX2 for one hour at room temperature. Nuclei were stained with 1 μg/ml 4’,6-diamidino-2-phenylindole (DAPI, Sigma, USA) for 5 minutes. For negative control, the samples were only treated with the secondary antibodies. All images were acquired by a camera (Eclipse 50i, Nikon, Japan) coupled to a fluorescence microscope (21). Then, the Image J software (V1.515) was utilized to evaluate, based on the intensity and the results which turned to be quantitative.

**Table 1:** Primers used for quantitative reverse transcription-polymerase chain reaction


Gene	Primer sequencing (5´-3´)	Product size

*YAP*	F: TAGCCCTGCGTAGCCAGTTA	177
	R: TCATGCTTAGTCCACTGTCTGT	
*CDX2*	F: GCAGAGCAAAGGAGAGAGGAAA	136
	R: AAGGGCTCTGGGACACTTCT	
*SOX2*	F: GGGAAATGGAAGGGGTGCAAAAGA	151
	R: TTGCGTGAGTGTGGATGGGATTGGT	
*OCT4*	F: CTGGGTTGATCCTCGGACCT	128
	R: CACAGAACTCATACGGCGGG	
*GATA3*	F: CCTCATTAAGCCCAAGCGA	185
	R: TGCCTTCCTTCTTCATAGTCAG	
*GAPDH*	F: CTCATTTCCTGGTATGACAACGA	119
	R: CTTCCTGTGCTCTTGCT	


### Statistical analysis

All experiments were performed using four independent biological replicates. Data were analysed using t test (for gene expression evaluation), chi-square (for protein expression and demographic information), or Mann Whitney tests (for comparison of the cycle numbers) using the SPSS statistical software (Ver. 16.0, IBM, USA(. Differences were considered significant at P<0.05.

**Table 2:** Patient characteristics in the young and old groups


Patient characteristics	Young	Old	Significance
	n=34	n=20	P value

Number of male factor	14 (45)	9 (45)	-
Number of female factor	1 (2)	0	-
Number of male and female fact	5 (14)	2 (5)	-
Number of recurrent abortion	1 (2)	2 (1)	-
Number of thalassemia	2 (8)	0	-
Number of sex determination	4 (11)	0	-
Number of unexplained	7 (17)	7 (35)	-
Number of agonist protocol	31 (91)	15 (75)	-
Number of antagonist protocol	3 (8)	5 (25)	-
Number of ICSI	19 (55)	8 (40)	-
Number of ICSI+IVF	15 (44)	12 (60)	-
Number of morula	38	17	-
Number of early blastocyst	10	8	-
Number of mid blastocyst	9	9	-
Number of expand blastocyst	18	8	-
Number of hatching blastocyst	7	3	-
Number of total oocyte	18.2 ± 7	11.6 ± 7	0.001
Number of GV	1.88 ± 1	2.44 ± 2	-
Number of Ml	1.44 ± 0.8	1.90 ± 1	-
Number of Mll	14.8 ± 7	8.76 ± 4	0.001
Mean BMI (Kg/m^2^)	26 ± 3	26.25 ± 4	-
Mean number of cycle	1.27 ± 0.5	2.55 ± 1.7	0.001
Female age (Y)	27 ± 2	38 ± 2	0.000
Male age (Y)	34 ± 1	42 ± 1	0.000


Data are presented as n (%) or mean ± SD. Significantly different at P<0.05. ICSI; Intracytoplasmic sperm injection, IVF; *In vitro* fertilization, GV; Germinal vesicle, MI; Metaphase I, MII; Metaphase II, and BMI; Body mass index.

## Results

### Patient demographic information

Table 2 shows that demographic information of the participants, total number of oocytes as well as MII oocytes in the young group was significantly higher than that of the old group (P<0.05). On the other hand, number of cycles in the young group was significantly lower than that of the old group (P<0.05). Additionally, mean age of the participants in these groups was significantly different (P<0.05). Based on the demographic data, it seems that ovarian resources begin to decline in women with >37 years of age.

### Quantitative reverse transcription-polymerase chain reaction analysis

**Fig.1:**
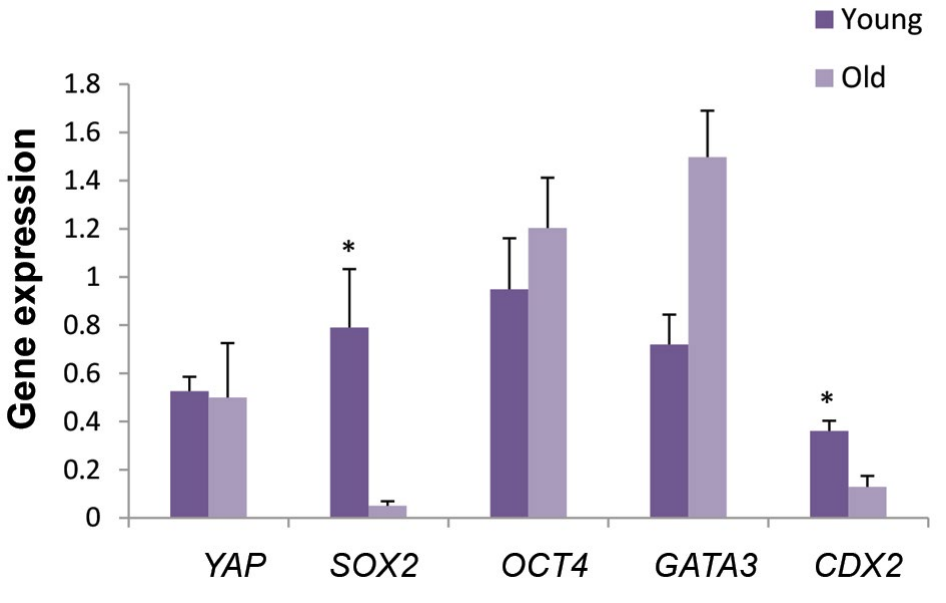
*YAP, SOX2, OCT4, CDX2* and *GATA3* gene expression levels in morula stage of the two groups. Data are presented as mean ± SE. *; Significant difference at P<0.05.

There was no significant difference between these two groups, in the expression of Hippo signaling marker (*YAP*) and pluripotency genes (*OCT4* and *GATA3*) at the Morula stage. However, *SOX2* and *CDX2* genes at the Morula stage had significantly higher expression levels in the young group, compared to the old group (P<0.05, Fig .1).

**Fig.2:**
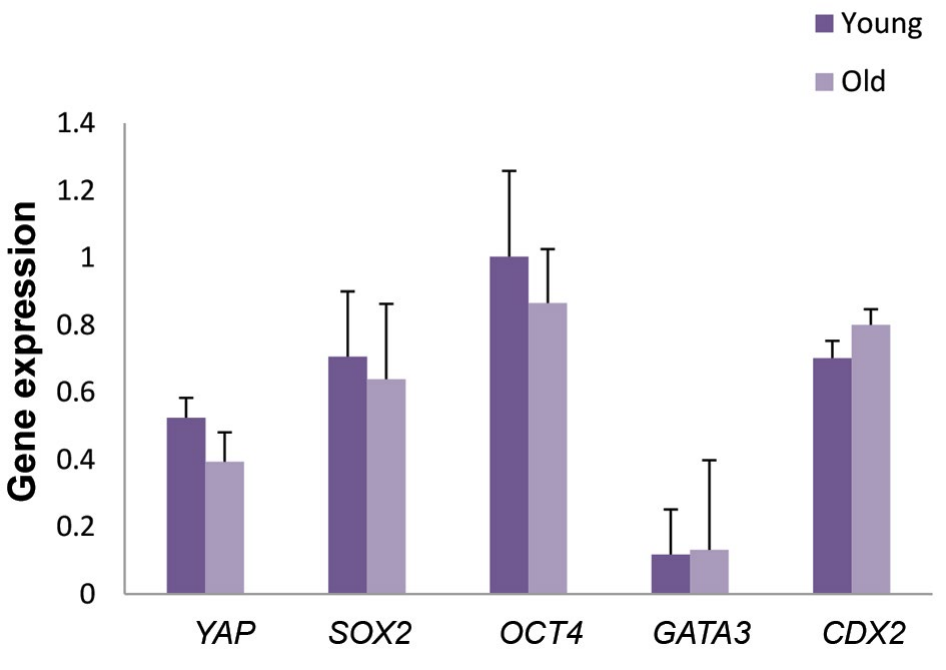
*YAP, SOX2, OCT4, CDX2* and *GATA3* gene expression levels in blastocyst stage of the two groups. Data are presented as mean ± SE.

There was no difference between these groups, in the expression of genes (*OCT4, CDX2, SOX2, GATA3* and *YAP*) at the blastocyst stage (Fig .2).

### Immunocytochemical analysis

Protein expression of OCT4, CDX2, YAP and phospho- YAP (p-YAP) at the blastocyst stage did not show significant variation, by comparing the young and old groups (Table 3).

**Fig.3:**
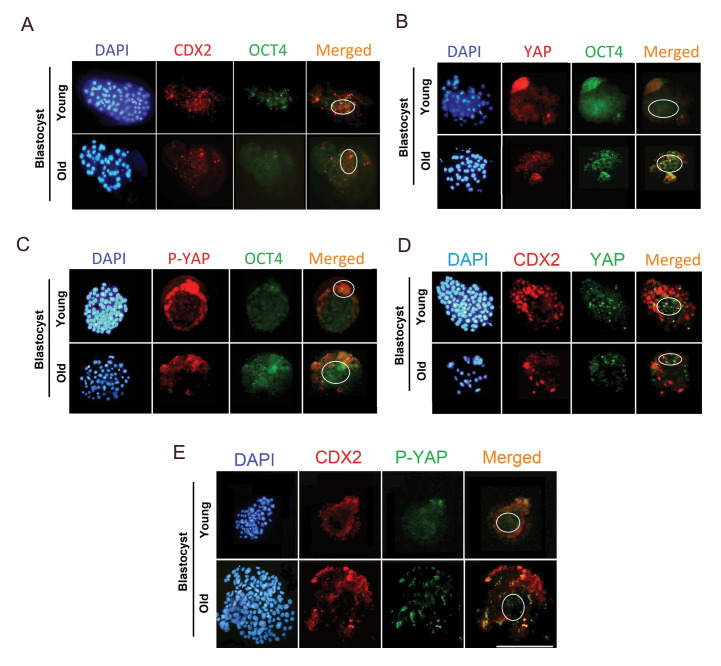
Immunofluorescence staining of developmental proteins (OCT4, CDX2, YAP and P-YAP) at the blastocyst stage. **A.** DAPI staining, immunofluorescence staining of CDX2 and OCT4 in the same cells as well as the merged DAPI and primary antibody-secondary antibody-FITC staining of CDX2 and OCT4 in blastocyst. **B.** DAPI staining, immunofluorescence staining of YAP and OCT4 in the same cells as well as the merged DAPI and primary antibody-secondary antibody-FITC staining of YAP and OCT4 in blastocyst. **C.** DAPI staining, immunofluorescence staining of P-YAP and OCT4 in the same cells as well as the merged DAPI and primary antibody-secondary antibody-FITC staining of P-YAP and OCT4 in blastocyst. **D.** DAPI staining, immunofluorescence staining of CDX2 and YAP in the same cells as well as the merged DAPI and primary antibody-secondary antibody-FITC staining of CDX2 and YAP in blastocyst. **E.** DAPI staining, immunofluorescence staining of CDX2 and P-YAP in the same cells as well as the merged DAPI and primary antibody-secondary antibody-FITC staining of CDX2 and P-YAP in blastocyst. ICM localization was distinguished by a circle (scale bars: 200 μm).

In the old group, at the blastocyst stage, P-YAP protein was found to be accumulated in the TE, but it was almost disappeared from the ICM. Additionally, contrary to the expectations, in the old group, YAP protein was expressed in the ICM rather than TE (Fig .3).

**Table 3:** Expression intensity of CDX2, OCT4, YAP and P-YAP proteins in the young and old groups


Proteins		Young group		Old group	

OCT4	CDX2	19.4 ± 2	22.1 ± 4	12.2 ± 5	10.4 ± 5
OCT4	YAP	31.8 ± 5	34.5 ± 3	13.8 ± 3	31 ± 2
OCT4	P-YAP	15.4 ± 1	35.4 ± 9	20.7 ± 4	24.9 ± 3
CDX2	YAP	41.6 ± 6	13.3 ± 1	30.18 ± 6	16.3 ± 2
CDX2	P-YAP	16.1 ± 2	24.3 ± 2	17.7 ± 6	27.7 ± 5


The data was evaluated based on the intensity. The signal intensities were quantified using Image J analysis software (V1.515). Data are presented as mean ± SD.

## Discussion

During differentiation of the blastomeres, cell polarity and position govern formation of TE and ICM (22). This process is mediated by various signalling pathways, such as Hippo signalling pathway which is the main regulator of cell growth, proliferation, differentiation and death (9, 14). In 2013, Lorthongpanich et al. (23) reported that inhibition of the components of Hippo signaling pathway disrupted the blastomeres differentiation into ICM. After LATS kinase decrease, they observed that *SOX2* and *OCT4* genes (ICM genes) were significantly reduced. On this basis, it can be said that Hippo pathway plays an important role in the early embryo differentiation by affecting these gene. Moreover, in they showed that *SOX2* and *CDX2* genes play an important role in development of embryos. *CDX2* gene is necessary for gastrulation. The mutation in this gene disturbs WNT, FGF and Hippo signalling. *SOX2* is initially expressed in the most of cells during morula stage. But, in the blastocyst stage, it is only expressed in ICM. Various factors can affect the embryo development from a zygote to a blastocyst, including quality of gametes, type of culture medium and oxygen concentration, as well as women’s age.

The infertility problems in 10% of women are associated with higher ages. As mentioned in the introduction, the best reproductive age for women is 20-30 years and women with >35 years show decrement of fertility potentiality (19). A study performed by Tehraninezhad et al. (24) in 2016, suggested age, as the most important underlying cause of infertility in women. Many of the infertility problems observed in older women are due to reduced ovarian reserve, producing fewer oocytes with lower quality. Several factors may reduce ovarian reserve, but one of the most important factors is the women‘s age. In this study, interesting results in terms of the expression of proteins were obtained by immunofluorescence staining. The location of YAP and the expression levels of P-YAP protein in the blastocyst embryos of the older group were not appropriate. In the older group, YAP protein expression was observed in TE cells, whereas in the blastocyst of women aged 20-30 years old, YAP was expressed in ICM. Furthermore, contrary to the expectation, expression of P-YAP in blastocyst collected from women aged >37 years old was observed in both ICM and TE cells. Therefore, with the age increase, it seems that Hippo pathway proteins are removed from their appropriate locations. According to the study performed by Hartley et al. (25) in 2015, if the protein processing step is not correctly taken after translation, the protein may be degraded by endopeptidase, thus negatively affecting the function or location of protein. To the best of our knowledge, there is no information in literature, regarding the gene expression of Hippo signaling pathway and its relationship with the women’s age in human embryo. In 2015, Li et al. (20) explored the Hippo signaling pathway and expression of *MVH/OCT4* genes in the mouse ovarian cortex. The level LATS2, MST1, MVH and *OCT4* were significantly decreased with increasing the age. YAP was also observed in the ovarian cortex of two-months old, but not 20-months old mice. In addition, YAP phosphorylation was found in ovarian cortex of seven-days old compared to 20-months old mice. Moreover, the amount of P-YAP/YAP was decreased, as 7-days old mice grew to 20 months. These results show that expressions of Hippo signaling pathway proteins are altered with increasing the age of ovary. A study performed by Pelissier et al. (21) in 2014 was conducted on breast epithelial progenitor cells and showed that biochemical, molecular and functional phenotypes differed before and after menopause. Moreover, Hippo pathway dysregulation affects differentiation and specificity of the cells. In previous studies, the expression of developmental and Hippo signaling pathway genes at the pre-implantation stage was decreased with increasing age in women. In the present study, no significant difference was observed in the expression of genes between these two groups, but immunofluorescent results showed that expression level of Hippo pathway proteins might be changed with increasing age.

This study has a few shortcomings which need to be considered in future studies. Our aim was to compare the gene expression of both young and old groups in two stages of morula and blastocyst, which required the same morphology. For this reason and according to the previous articles which have been done, total mRNA was extracted (23).

## Conclusion

The result of this study showed that although the levels of *YAP* and *P-YAP* gene expression were not significantly different between young and old groups, in blastocyst stage, P-YAP protein was found to be accumulated in the TE and it was almost disappeared from ICM. Contrary to expectations, in the old (women with more than 37 years old) group, YAP protein expression was expressed in ICM rather than TE. These data may indicate the inappropriate functionality of Hippo signaling pathway at the advanced ages. More accurate results could be obtained, if immunestaining was performed for all four proteins in the embryo.

## References

[1] Bleil JD, Wassarman PM (1980). Mammalian sperm-egg interaction: identification of a glycoprotein in mouse egg zonae pellucidae possessing receptor activity for sperm. Cell.

[2] Florman HM, Storey BT (1982). Mouse gamete interactions: the zona pellucida is the site of the acrosome reaction leading to fertilization in vitro. Dev Biol.

[3] Oron E, Ivanova N (2012). Cell fate regulation in early mammalian development. Phys Biol.

[4] Tarkowski AK, Wróblewska J (1967). Development of blastomeres of mouse eggs isolated at the 4-and 8-cell stage. Development.

[5] Takaoka K, Hamada H (2012). Cell fate decisions and axis determination in the early mouse embryo. Development.

[6] Dietrich J-E, Hiiragi T (2007). Stochastic patterning in the mouse pre-implantation embryo. Development.

[7] Zernicka-Goetz M, Morris SA, Bruce AW (2009). Making a firm decision: multifaceted regulation of cell fate in the early mouse embryo. Nat Rev Genet.

[8] Yamanaka Y, Lanner F, Rossant J (2010). FGF signal-dependent segregation of primitive endoderm and epiblast in the mouse blastocyst. Development.

[9] Yu F-X, Zhao B, Guan K-L (2015). Hippo pathway in organ size control, tissue homeostasis, and cancer. Cell.

[10] Reddy BVVG, Irvine KD (2013). Regulation of Hippo signaling by EGFRMAPK signaling through Ajuba family proteins. Dev Cell.

[11] Sasaki H (2017). Roles and regulations of Hippo signaling during preimplantation mouse development. Dev Growth Differ.

[12] Cockburn K, Biechele S, Garner J, Rossant J (2013). The Hippo pathway member Nf2 is required for inner cell mass specification. Curr Biol.

[13] Yu F-X, Guan K-L (2013). The Hippo pathway: regulators and regulations. Genes Dev.

[14] Varelas X (2014). The Hippo pathway effectors TAZ and YAP in development, homeostasis and disease. Development.

[15] Nishioka N, Inoue K, Adachi K, Kiyonari H, Ota M, Ralston A (2009). The Hippo signaling pathway components Lats and Yap pattern Tead4 activity to distinguish mouse trophectoderm from inner cell mass. Dev Cell.

[16] Lorthongpanich C, Issaragrisil S (2015). Emerging role of the Hippo signaling pathway in position sensing and lineage specification in mammalian preimplantation embryos. Biol Reprod.

[17] Wicklow E, Blij S, Frum T, Hirate Y, Lang RA, Sasaki H (2014). Hippo pathway members restrict SOX2 to the inner cell mass where it promotes ICM fates in the mouse blastocys. PLoS Genet.

[18] Mantikou E, Jonker MJ, Wong KM, van Montfoort APA, de Jong M, Breit TM (2015). Factors affecting the gene expression of in vitro cultured human preimplantation embryos.Hum Reprod.

[19] Bellieni C (2016). The best age for pregnancy and undue pressures. J Fam Reprod Health.

[20] Li J, Zhou F, Zheng T, Pan Z, Liang X, Huang J (2015). Ovarian germline stem cells (OGSCs) and the Hippo signaling pathway association with physiological and pathological ovarian aging in mice. Cell Physiol Biochem.

[21] Pelissier FA, Garbe JC, Ananthanarayanan B, Miyano M, Lin C, Jokela T (2014). Age-related dysfunction in mechanotransduction impairs differentiation of human mammary epithelial progenitors. Cell Rep.

[22] Manzanares M, Rodriguez TA (2013). Development: Hippo signalling turns the emberyo inside out. Current Biology.

[23] Lorthongpanich C, Messerschmidt DM, Chan SW, Hong W, Knowles BB, Solter D (2013). Temporal reduction of LATS kinases in the early preimplantation embryo prevents ICM lineage differentiation. Genes Dev.

[24] Tehraninezhad ES, Mehrabi F, Taati R, Kalantar V, Aziminekoo E, Tarafdari A (2016). Analysis of ovarian reserve markers (AMH, FSH, AFC) in different age strata in IVF/ICSI patients. Int J Reprod Biomed.

[25] Hartley AM, Zaki AJ, McGarrity AR, Robert-Ansart C, Moskalenko AV, Jones GF (2015). Functional modulation and directed assembly of an enzyme through designed non-natural post-translation modification. Chem Sci.

